# Dr. Edgar Rose Rust

**Published:** 1904-09

**Authors:** 


### Obituary.

### DR. EDGAR ROSE RUST.

Died, Thursday, June 2, 1904, at Coronado Beach, Cal., Dr. Edgar Rose Rust, in the forty-fifth year of his age. Dr. Rust had recently left his home in Denver, Col., and was visiting, with his family, places of interest on the coast of California, when an acute middle-ear disease, followed by erysipelas, caused his death. Dr. Rust was the son of John and Elizabeth E. Rust, and was born at Waverly, Westmoreland County, Va., which for more than two hundred years was the home of his paternal ancestors, who were of English origin. In early life Dr. Rust acquired a good education through private tutors at home, and later in the schools of the neighboring city of Baltimore. Soon after leaving school he entered upon the study of dentistry under the perceptorship of his brother, Dr. David N. Rust, of Washington, D. C., then located in Alexandria, Va. He graduated from the Baltimore College of Dental Suregry in the year 1882, and shortly thereafter became associated on very favorable terms with the court dentist of Madrid, Spain. This association continued but a few months, when the death of the latter left Dr. Rust under the disadvantages of a brief acquaintance with his associate's clientele and a limited command of the language of the country; but these disadvantages soon disappeared and a lucrative practice was readily acquired. The climate of Madrid was, however, found so unsuited to Dr. Rust that he was compelled to relinquish his promising professional career there and return, in 1885, to the capital of his native country, where he was again successful in establishing a large practice among the more desirable of the resident and official classes of Washington. But more than mere business success, which he seemed quite able to attain anywhere in the world, Dr. Rust's genial and sociable nature found a greater degree of gratification through the large measure of social advantages which he enjoyed at the nation's capital. He was an honored and active member of the old Washington City Dental Society, and was regarded by its members as an exceptionally skilful operator, having been one of the few who operated with equal facility according to the higher ideals of both the cohesive and non-cohesive gold advocates. Among his numerous Washington friends were numbered the best type of his own and other professions, and the trial of his life came when, acting on the advice of his physicians, he was compelled to sever these relations and seek to maintain health in a more suitable climate. It was under such circumstances that he resigned the presidency of the Washington City Dental Society in 1891, gave up his social and professional connections in Washington, and removed to Denver, Col., where he continued in professional labors for three years, when he retired from active practice and spent his remaining years in country life near Denver. While he continued actively and successfully several business enterprises, he took an especial pride in owning and raising fine stock, some of his horses being among the most valuable in America.

Dr. Rust married, in 1894, Miss Stella Hoyt, a member of an old and highly respected Denver family. His wife and their three children survive him.

At a meeting of the District of Columbia Dental Society, formerly the Washington City Dental Society, held June 21, 1904, a resolution was passed instructing the undersigned committee to furnish the dental journals with the above sketch and to convey to Dr. Rust's family suitable expressions of the Society's sympathy and sorrow.

Williams Donnally,
W. E. Pairo,
G. F. Simpson,
*Committee.*

